# Biochemical and Molecular Characterization of a Serine Keratinase from *Brevibacillus brevis* US575 with Promising Keratin-Biodegradation and Hide-Dehairing Activities

**DOI:** 10.1371/journal.pone.0076722

**Published:** 2013-10-11

**Authors:** Nadia Zaraî Jaouadi, Hatem Rekik, Abdelmalek Badis, Sahar Trabelsi, Mouna Belhoul, Amina Benkiar Yahiaoui, Houda Ben Aicha, Abdessatar Toumi, Samir Bejar, Bassem Jaouadi

**Affiliations:** 1 Laboratory of Microorganisms and Biomolecules (LMB), Centre of Biotechnology of Sfax (CBS), University of Sfax, Sfax, Tunisia; 2 Laboratory of Natural Products Chemistry and Biomolecules (LNPCB), University of Saàd Dahlab (USD) of Blida, Blida, Algeria; 3 National Centre for Research and Development of Fisheries and Aquaculture (CNRDPA), Bousmail (W. Tipaza), Algeria; 4 National Leather and Shoe Center (CNCC), Mégrine, Ben Arous, Tunisia; Moffitt Cancer Center, United States of America

## Abstract

Dehairing is one of the highly polluting operations in the leather industry. The conventional lime-sulfide process used for dehairing produces large amounts of sulfide, which poses serious toxicity and disposal problems. This operation also involves hair destruction, a process that leads to increased chemical oxygen demand (COD), biological oxygen demand (BOD), and total suspended solid (TSS) loads in the effluent. With these concerns in mind, enzyme-assisted dehairing has often been proposed as an alternative method. The main enzyme preparations so far used involved keratinases. The present paper reports on the purification of an extracellular keratinase (KERUS) newly isolated from *Brevibacillus brevis* strain US575. Matrix assisted laser desorption ionization-time of flight mass spectrometry (MALDI-TOF/MS) analysis revealed that the purified enzyme was a monomer with a molecular mass of 29121.11 Da. The sequence of the 27 N-terminal residues of KERUS showed high homology with those of *Bacillus* keratinases. Optimal activity was achieved at pH 8 and 40°C. Its thermoactivity and thermostability were upgraded in the presence of 5 mM Ca^2+^. The enzyme was completely inhibited by phenylmethanesulfonyl fluoride (PMSF) and diiodopropyl fluorophosphates (DFP), which suggests that it belongs to the serine protease family. KERUS displayed higher levels of hydrolysis, substrate specificity, and catalytic efficiency than NUE 12 MG and KOROPON® MK EG keratinases. The enzyme also exhibited powerful keratinolytic activity that made it able to accomplish the entire feather-biodegradation process on its own. The *kerUS* gene encoding KERUS was cloned, sequenced, and expressed in *Escherichia coli*. The biochemical properties of the extracellular purified recombinant enzyme (rKERUS) were similar to those of native KERUS. Overall, the findings provide strong support for the potential candidacy of this enzyme as an effective and eco-friendly alternative to the conventional chemicals used for the dehairing of rabbit, goat, sheep and bovine hides in the leather processing industry.

## Introduction

Leather making is an important socio-economic activity for several countries throughout the world. This industry is, however, associated with severe health and ecological problems owing to the use of various chemicals and release of hazardous effluents in the environment. Leather processing involves a complex set of steps, from skin to finished product, including soaking, dehairing, bating, and tanning. These operations involve the application of materials that are capable of degrading proteinaceous matter present in the hides and skins.

The conventional methods of leather processing involve the application of various hazardous chemicals, notably sodium sulfide, which generates several environmental and waste disposal problems. In order to overcome the hazards caused by these effluents, enzymes have often been proposed as viable alternatives. In fact, enzymes have long been used as alternatives to chemicals to improve the efficiency and cost-effectiveness of a wide range of industrial systems and processes [1]. Proteases have particularly been reported to constitute a resourceful class of enzymes with promising industrial applications. Of special interest, keratinases (E.C. 3.4.21/24/99.11) are a group of metallo or serine proteinases that can degrade the insoluble structure forming keratin substrates. Keratins are a class of fibrous, insoluble and abundant structural proteins that constitute the major components of structures growing from the skin of vertebrates, such as hair, wool, nails, hooves, horns and feather quills. Due to their high degree of cross-linking to disulphide bonds, hydrogen bonds, and hydrophobic interactions, these proteins show high stability and resistance to proteolytic enzymes, including trypsin, pepsin, and papain [2]. In enzymatic catalysis, the disulfide bonds of keratin are reduced by disulfide reductase followed by the action of keratinases, which simultaneously degrade the keratin into oligo- and monomeric products [3].

The use of proteolytic enzymes as alternatives to chemicals has brought significant improvements in terms of the efficiency, cost-effectiveness and eco-friendliness of the leather processing industry. Proteases are used for the selective hydrolysis of non-collagenous compounds and removal of non-fibrillar proteins such as albumins and globulins. The purpose of soaking is to swell the hide, and this step was traditionally performed using alkali. Currently, microbial alkaline proteases are used to ensure a faster absorption of water and reduce the time required for soaking. The use of nonionic and, to some extent, anionic surfactants is compatible with the use of enzymes. The conventional method of dehairing and dewooling involves the development of an extremely alkaline condition followed by treatment with sulfide to solubilize the protein matter at the hair base. The fundamental aims of dehairing are the detachment of hair skin and opening up of collagen fibrous structure. The methods currently employed for dehairing are based on the application of alkaline proteases, hydrated lime, and sodium chloride, which have contributed enormously to the reduction of the amounts of wastewater generated [4]. The conventional lime-sulfide process is, however, known to generate large amounts of sulfide, which poses serious health and waste disposal problems. It also leads to the destruction of the hair, thus causing high COD, BOD, and TSS loads in the effluent. The search for cleaner technologies that can help overcome the serious problems associated with the conventional dehairing methods has, therefore, become a necessity in the leather industry [5].

Furthermore, early bating methods relied on animal feces as a source of proteases, which were later replaced by pancreatic trypsin. The latter is currently employed in combination with other *Bacillus* and *Aspergillus* neutral proteases. In fact, while the selection of the enzyme species depends on its specificity for matrix proteins, such as elastin and keratin, the specification of the amount of enzyme needed depends on the type of leather (soft or hard) to be produced. Overall, the introduction of enzymes to the dehairing and bating processes has not only alleviated the serious concerns associated with the leather making industry but have also contributed to the energy saving practices worldwide [6]. Three different proteases, namely Aquaderm, NUE (Novozymes A/S, Danemark), and KOROPON (MK Michael Kors leathers, Brazil), are currently manufactured for use in soaking, dehairing, and bating, respectively.

Due to their attractive properties and attributes, keratinases have been isolated from various microorganisms and introduced into a wide range of biotechnological applications, including those in the feed, fertilizer, detergent, leather and pharmaceutical industries [3]. Keratinases/keratinolytic microorganisms have, for instance, been in use for the production of feather-meal, the enhancement of drug delivery, the hydrolysis of prion, the construction of biodegradable films, and the production of biofuels [7]. They have been purified from diverse microorganisms, including fungi, such as *Purpureocillium lilacinum* LPS # 876 [8] and *Chryseobacterium gelum* [9], and bacteria, such as *Streptomyces* [10] and *Bacillus* [11], [12]. In fact, several serine peptidases have so far been isolated, purified and characterized from various species, including *B. pumilus* CBS [13], *B. subtilis* [14], and *B. circulans* DZ100 [15]. The biochemical and molecular characteristics of keratinases have also been extensively investigated in the scientific literature. The use of keratinolytic bacteria to be appropriate for the production of feather hydrolysates has been the subject of some patented literature processes [16]–[19], and the keratinase from *B. licheniformis* PWD-1 is commercially produced under the trade name Versazym.

Despite the large flow of data on keratinases, and to the authors’ knowledge, no previous work has so far been performed to investigate the keratinase producing potential of the *Brevibacillus brevis* keratinase family. In fact, the use of *Bacillus* enzymes for large-scale applications is still very limited by their relatively low stabilities and catalytic activities under the operational conditions required for the dehairing process, namely moderate temperature and neutral/alkali pH values as well as the presence of denaturing agents. The isolation and screening of new keratinolytically active *Bacillus* strains from natural habitats or neutral/alkaline wastewater could, therefore, open new opportunities for the discovery and use of novel keratinases for application in poultry and leather processing industries. Accordingly, the present study aimed to report on the purification and biochemical characterization of a novel keratinolytic enzyme (KERUS) from *Br. brevis* strain US575 isolated from contaminated soil samples collected from a local leather tannery (M’Saken-Sousse, Tunisia). The nucleotide and amino acid sequences, cloning, and expression of the encoding gene (*kerUS*) were also determined.

## Materials and Methods

### 2.1. Substrates, Chemicals, and Commercial Leather Enzymes

Unless specified otherwise, all substrates, chemicals, and reagents were of the analytical grade or highest available purity, and were purchased from Sigma Chemical Co. (St. Louis, MO, USA). NUE 12 MG, a commercial proteolytic/elastolytic enzyme formulation supplied by Novozymes A/S (Bagsvaerd, Denmark), is a protease produced by the submerged fermentation of a genetically modified *Bacillus* for leather dehairing. KOROPON® EG, a commercial proteolytic enzyme formulation supplied by KOROPON MK-Brazil, is a protease of pancreatic origin with deliming salts produced by the submerged fermentation of a genetically modified *Bacillus* for leather bating, and was kindly provided by the SO. SA. CUIR leather tannery (M’Saken, Sousse, Tunisia).

### 2.2. Isolation, Culture and Growth Conditions of Keratinase-Producing Microorganisms

Soil samples were collected from the contaminated soil of the private SO. SA. CUIR leather tannery at M’Saken City (Sousse, Tunisia) to isolate keratinase-producing microorganisms. The soil samples collection was carried out on the private land owned to the leather tannery and the study on this site was conducted with the permission from the private property owner of the company. The samples were dispersed in sterile distilled water and heated for 30 min at 80°C to kill vegetative cells. They were then plated onto chicken feather-meal agar plates containing (g.l^−1^): chicken feather-meal, 5; soy peptone, 2; yeast extract, 3; and bacteriological agar, 20 at pH 7.4. The plates were incubated at 37°C over night to obtain colonial growth. The colonies with clear zones formed by the hydrolysis of keratin were evaluated as keratinase producers. Several keratinolytic strains were isolated, and strain US575, which exhibited a large clear zone of hydrolysis, was selected for further experimental work.

The growth medium used for keratinase production by strain US575 at pH 7.4 consisted of (g.l^−1^): chicken feather-meal, 10; soy peptone, 5; (NH_4_)_2_SO_4_, 2; MgSO_4_·7H_2_O, 1; CaCl_2_, 5; K_2_HPO_4_, 1; KH_2_PO_4_, 1; NaCl, 5; and trace elements 2% (v/v) [composed of (g.l^−1^): ZnCl_2_, 0.4; FeSO_4_·7H_2_O, 2; H_3_BO_3_, 0.065; and MoNa_2_O_4_·2H_2_O, 0.135]. The Media were autoclaved for 20 min at 121°C. Cultivations were performed in 1,000 ml conical flasks with a working volume of 100 ml for 72 h at 37°C and 250 rpm on a rotary shaker. Growth kinetics were monitored by measuring absorbance at 600 nm. The cell-free supernatant was recovered by centrifugation (9,000×*g*, 30 min) at 4°C, and served as keratinase preparation in subsequent studies.

### 2.3. Identification of the *Bacillus* Strain and Phylogenetic Analysis

Analytical profiling index (API) strip tests and 16S rRNA gene sequencing (ribotyping) were carried out for the identification of the genus to which the strain belonged. API 50 CH strips (bioMérieux, SA, Marcy-l’Etoile, France) were used to investigate the physiological and biochemical characteristics of strain US575 following the manufacturer’s instructions. The 16S rRNA gene was amplified by polymerase chain reaction (PCR) using forward primer, F-73, 5′-AGAGTTTGATCCTGGCTCAG-3′, and reverse primer, R-74, 5′-AAGGAGGTGATCCAAGCC-3′, designed from the conserved zones within the rRNA operon of *E. coli* [20].

The genomic DNA of strain US575 was purified using the Wizard® Genomic DNA Purification Kit (Promega, Madison, WI, USA) and then used as a template for PCR amplification (35 cycles, 94°C for 30 s denaturation, 65°C for 60 s primer annealing, and 72°C for 120 s extension). The amplified ∼1.5 kb PCR product was cloned in the pGEM-T Easy vector (Promega, Madison, WI, USA), leading to pUS1 plasmid (this study). The *E. coli* DH5α [F^−^ *supE44 Φ80 δlacZ ΔM15 Δ(lacZYA*-*argF) U169 endA1 recA1 hsdR17 (r_k_* ^−^, *m_k_^+^) deoR thi*-*1 λ^−^ gyrA96 relA1*] (Invitrogen, Carlsbad, CA, USA) was used as a host strain. All recombinant clones of *E. coli* were grown in LB broth media with the addition of ampicillin, isopropyl-thio-β-D-galactopyranoside (IPTG), and X-gal for screening. DNA electrophoresis, DNA purification, restriction, ligation, and transformation were all performed according to the method previously described by Sambrook et al. [21].

Phylogenetic and molecular evolutionary analyses were conducted *via* the molecular evolutionary genetics analysis (MEGA) software version 4.1. Distances and clustering were calculated using the neighbor-joining method. Bootstrap analysis was used to evaluate the tree topology of the neighbor-joining data by performing 100 re-samplings.

### 2.4. Enzyme Assays

Keratinolytic activity was determined using keratin azure or azo-casein as a substrate [22]. Unless otherwise stated, 1 ml of 10 g.l^−1^ keratin azure, suspended in 100 mM 4-(2-hydroxyethyl)-1-piperazineethanesulfonic acid (HEPES) buffer and supplemented with 5 mM CaCl_2_ at pH 8 (Buffer A), was mixed with 1 ml of a suitably diluted enzyme solution. The sample was incubated with shaking for 30 min at 40°C and 250 rpm. The assay mixture was cooled in ice-cold water for 5 min. The unutilized substrates were removed by centrifugation (10,000×*g*, 20 min) and filtration through Millipore cellulose filters (0.45 µm). The released azo dye was measured in the filtrate at 440 nm, and activity was expressed in keratin units (KU). The control consisted of enzyme and buffer without substrate. One KU was defined as the amount of enzyme causing an increase of 0.1 in absorbance at 440 nm in one min under the experimental conditions described.

Caseinolytic activity was measured using the Folin-Ciocalteu method and as previously described elsewhere [23] with Hammersten casein (Merck, Darmstadt, Germany), keratin, elastin-orcein gelatin, hemoglobin, myoglobin, or albumin as a substrate. One casein unit (CU) was defined as the amount of enzyme that hydrolyzed the substrate and that produced 1 µg of amino acid equivalent to tyrosine per min at 40°C and pH 8 in buffer A.

Disulfide bond-reducing activity was determined at 412 nm by measuring the yellow-colored sulfide formed upon the reduction of 5,5′-dithio-bis-2-nitro benzoic acid (DTNB) as described by Jaouadi et al. [24]. One unit of disulfide bond-reducing activity (DU) was defined as the amount of enzyme that catalyzed the formation of 1 µmole of sulfide per min.

### 2.5. Enzyme Purification

Five hundred ml of a 28-h old culture of *Br. brevis* strain US575 was centrifuged for 30 min at 9,000×*g* to remove microbial cells. The supernatant containing extracellular keratinase was used as the crude enzyme preparation and was submitted to the following purification steps. The supernatant was precipitated between 50% and 70% ammonium sulfate saturation. The precipitate was then recovered by centrifugation at 12,000×*g* for 30 min, resuspended in a minimal volume of 50 mM HEPES buffer containing 5 mM CaCl_2_ and 20 mM NaCl at pH 7.5 (Buffer B), and dialyzed overnight against repeated changes of buffer B. Insoluble material was removed by centrifugation at 12,000×*g* for 30 min. The supernatant was loaded and applied to a high performance liquid chromatography (HPLC) system using a Bio-Sil SEC 125-5 Column (7.8 mm×300 mm, Bio-Rad Laboratories, Inc., Hercules, CA, USA) that was pre-equilibrated with buffer B. Proteins were separated by isocratic elution at a flow rate of 30 ml.h^−1^ with buffer C and detected using a UV-VIS Spectrophotometric detector (Knauer, Berlin, Germany) at 280 nm. The fractions containing keratinase activity (eluted at a void volume of 1.7, with retention time of 18 min) were pooled and then applied to a Mono Q Sepharose column (Pharmacia, Uppsala, Sweden) equilibrated with 50 mM 2-(*N*-morpholino) ethanesulfonic acid (MES) buffer containing 5 mM CaCl_2_ at pH 6 (Buffer C). The column was rinsed with 500 ml of the same buffer. Adsorbed material was eluted with a linear NaCl gradient (0 mM to 500 mM) in buffer C at a rate of 40 ml.h^−1^. The column (2.6 cm×20 cm) was extensively washed with buffer C until the optical density of the effluent at 280 nm was zero. Fractions of 5 ml each were collected at a flow rate of 40 ml.h^−1^ and analyzed for keratinolytic activity and protein concentration. Keratinase activity was eluted between 140 mM and 240 mM NaCl. Pooled fractions containing keratinase activity were concentrated in centrifugal micro-concentrators (Amicon Inc., Beverly, MA, USA) with 10-kDa cut-off membranes and were stored at −20°C in a 20% glycerol (v/v) solution for further analysis.

### 2.6. Determination of Protein Concentration and Analytical Methods

Protein concentration was determined by the method of Bradford [25] using a Dc protein assay kit purchased from Bio-Rad Laboratories (Hercules, CA, USA), with bovine serum albumin (BSA) as a reference. The analytical polyacrylamide gel electrophoresis of proteins in the presence of sodium dodecyl sulfate (SDS-PAGE) was performed following the method of Laemmli [26]. The protein bands were visualized with Coomassie Brilliant Blue R-250 (Bio-Rad Laboratories, Inc., Hercules, CA, USA) staining. Keratin azure zymography staining was performed as previously described by Jaouadi et al. [10]. The molecular mass of purified KERUS was analyzed in linear mode by MALDI-TOF/MS using a Voyager DE-RP instrument (Applied Biosystems/PerSeptive Biosystems, Inc., Framingham, MA, USA). Data was collected with a Tektronix TDS 520 numeric oscillograph and analyzed using the GRAMS/386 software (Galactic Industries Corporation, Salem, NH, USA). Bands of purified KERUS on SDS gels were transferred to a ProBlott membrane (Applied Biosystems, Foster City, CA, USA), and N-terminal sequence analysis was performed by automated Edman’s degradation using an Applied Biosystem Model 473A gas-phase sequencer. The N-terminal sequence was compared to those in the Swiss-Prot/TrEMBL database using the BLAST homology search program (NCBI, NIH, USA).

### 2.7. Effects of Inhibitors and Metal Ions on Keratinase Stability

The effects of PMSF, DFP, soybean trypsin inhibitor (SBTI), benzamidine hydrochloride hydrate, *Nα*-*p*-tosyl l-phenylalanine chloromethyl ketone (TPCK), *Nα-p*-tosyl l-lysine chloromethyl ketone (TLCK), DTNB, monoiodoacetic acid (MIA), ld-dithiothreitol (DTT), 2-mercaptoethanol (2-ME), *N*-ethylmalemide (NEM), leupeptin hemisulfate salt, pepstatin A, 1,2-epoxy-3-(*p*-nitrophenoloxy) propane (EPNP), EDTA, EGTA, and various monovalent and divalent metal ions (5 mM) on keratinase stability were investigated by pre-incubating the purified KERUS enzyme for 1 h at room temperature with each inhibitor, and for 1 h at 40°C in the presence of metal ions. Enzyme assays were carried out under standard assay conditions.

### 2.8. Effects of pH and Temperature on Keratinase Activity and Stability

The activity of the KERUS, NUE, and KOROPON enzymes were measured at a pH range of 2 to 13 at 40°C using keratin azure as a substrate. Their pH stability was determined by pre-incubation in buffer solutions at 40°C and different pH values for 72 h. Aliquots were withdrawn, and residual enzymatic activity was determined at pH 8 and 40°C. The following buffer systems, supplemented with 5 mM CaCl_2_, were used at 100 mM: glycine-HCl for pH 2–5, MES for pH 5–6; HEPES for pH 6–8, Tris-HCl for pH 8–9, glycine-NaOH for pH 9–11, bicarbonate-NaOH for pH 11–11.5, disodium hydrogen phosphate-NaOH for pH 11.5–12, and potassium chloride-NaOH for pH 12–13.

The effect of temperature on the enzyme activities of KERUS, NUE, and KOROPON were examined at 20–80°C and pH 8 for 20 min. Their thermal stability was determined by incubation at 30–60°C and pH 8 for 72 h in the presence and absence of 5 mM CaCl_2_. Aliquots were withdrawn at specific time intervals to test remaining activity under standard conditions. The non-heated enzyme, which was cooled on ice, was considered as a control (100%).

### 2.9. Effect of Substrate Specificity and Kinetic Measurements

The substrate specificity of KERUS was determined using natural (keratin, elastin, gelatin, casein, hemoglobin, myoglobin, and albumin) and modified (keratin azure, azo-casein, and collagen types I and II: FITC conjugate) protein substrates as well as ester [*N*-benzol-l-arginine ethyl ester (BAEE), *N*-benzol-l-tyrosine ethyl ester (BTEE), *S*-benzyl-l-cysteine ethyl ester hydrochloride (BCEE), and *N*-acetyl-l-tyrosine ethyl ester monohydrate (ATEE)] and synthetic peptide [*N*-succinyl-l-Tyr-l-Leu-l-Val-*p*-nitroanilide, *N*-succinyl-l-Ala-l-Ala-l-Phe-*p*-nitroanilide, *N*-succinyl-l-Ala-l-Ala-l-Ala-*p*-nitroanilide, *α*-benzoyl-l-tyrosine *p*-nitroanilide (BAPNA), *N*-succinyl-l-Ala-l-Ala-l-Val-*p*-nitroanilide, *N*-succinyl-l-Ala-l-Ala-l-Val-l-Ala-*p*-nitroanilide, *N*-methoxysuccinyl-l-Ala-l-Ala-l-Pro-l-Val-*p*-nitroanilide, *N*-succinyl-l-Ala-l-Ala-l-Pro-l-Met-*p*-nitroanilide, *N*-succinyl-l-Ala-l-Ala-l-Pro-l-Phe-*p*-nitroanilide, *N*-succinyl-l-Ala-l-Ala-l-Pro-l-Leu-*p*-nitroanilide, glutaryl-l-Ala-l-Ala-l-Pro-l-Leu-*p*-nitroanilide, and *N*-succinyl-l-Ala-l-Pro-l-Ala-*p*-nitroanilide] substrates. Enzymatic activities were determined on each substrate according to standard conditions.

Kinetic parameters were calculated from the initial activity rates of the purified enzymes (KERUS, NUE, and KOROPON) using natural (keratin) and modified (keratin azure) proteins and ester (BAEE) and synthetic peptide [*N*-succinyl-l-Tyr-l-Leu-l-Val-*p*-nitroanilide] substrates. The pH and temperature values used in the kinetic study were adjusted to the optimum conditions for each enzyme (KERUS, pH 8, 40°C; NUE, pH 9, 50°C; and KOROPON, pH 7.5, 35°C). For the natural substrate, one keratin unit was defined as the amount of enzyme that hydrolyzed the substrate and that produced 1 µg of amino acid equivalent to tyrosine per min at 660 nm under the assay conditions used. For the modified substrate, one keratin azure unit was defined as the amount of enzyme causing an increase of 0.1 in absorbance at 440 nm in one min under the experimental conditions described. For the ester substrate, the rate of change in absorbance at 253 nm was measured for 3 min. One BAEE unit was defined as the amount of enzyme causing an absorbance change rate of 0.001 per min under the assay conditions. For the synthetic peptide substrate, the amount of released *p*-nitroanilide (*p*-NA) was recorded at 410 nm. One unit of enzymatic activity was defined as the amount of enzyme releasing 1 µmole of *p*-NA under standard assay conditions. The reaction was performed at different substrate concentrations ranging from 0.05 mM to 50 mM, for 15 min in assay buffer supplemented with 10% (v/v) dimethyl sulphoxide and 1% (v/v) Triton X-100. Each assay was carried out in triplicate, and kinetic parameters were estimated by Lineweaver–Burk plots. Kinetic constants, Michaelis–Menten constant (*K*_m_) and maximal reaction velocity (*V*_max_) values were calculated using the Hyper32 software package developed at Liverpool University (http://hompage.ntlword.com/john.easterby/hyper32.html). The value of the turnover number (*k*_cat_) was calculated using the following equation
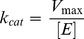
where [*E*] refers to the active enzyme concentration and *V*_max_ to maximal velocity.

### 2.10. Determination of Hydrolysis Degree

Keratin hydrolysis was carried out at 40°C and pH 8 (for KERUS); 50°C and pH 9 (for NUE); and 35°C and pH 7.5 (for KOROPON). The pH was kept constant throughout hydrolysis by adding NaOH 4 N. An amount of 5 g of chicken feather-meal was dissolved in 100 ml of assay buffer and then treated with 2,000 U.ml^−1^ of the purified enzymes, namely KERUS, NUE, and KOROPON. The NaOH amount needed to maintain the pH constant was proportional to the degree of hydrolysis (DH). Enzymatic reactions were stopped when the DH became constant. The DH, defined as the percent ratio of the number of peptide bonds broken (*h*) to the total number of peptide bonds in the assayed substrate (*h_tot_*), was calculated for each case from the amount of the base (NaOH) added to keep the pH constant during hydrolysis [12], [27] as given below
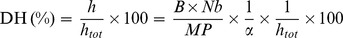
where *B* refers to the amount of the base consumed (ml) to keep the pH constant during the reaction, *Nb* to the normality of the base, *MP* to the mass (g) of protein (N×6.25), and *α* to the average dissociation degree of the *α*-NH_2_ groups released during hydrolysis expressed as



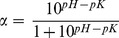
where *pH* and *pK* refer to the values at which the proteolysis was performed. The total number of peptide bonds (*h_tot_*) in the feather-meal protein concentrate was assumed to be 8.1 meq.g^−1^ [12], [27].

### 2.11. Determination of Keratin Biodegradation

The keratin-biodegradation ability of *Br. brevis* strain US575 was also investigated using chicken feather-meal, chicken feather, rabbit hair, goat hair, bovine hair, and sheep wool as keratinacious materials. All keratin substrates were freshly obtained from a local slaughterhouse (Sfax municipal slaughterhouse, permission was obtained from this slaughterhouse to use these animal parts), rinsed to remove excess blood, and autoclaved to sterilize. Feather-meal was obtained as previously described by Jaouadi et al. [24]. The substrates were then separately shake-incubated to the medium at 1% (w/v) for 28 h at different temperatures ranging from 30°C to 45°C, under shake culture condition with 3.5×10^7^ cells ml^−1^ as an initial inoculum density. Protein concentration was measured as described by the Bradford’s method [25]. The keratin in the cultures was harvested by filtration through Whatman No. 3 filter paper, washed twice with distilled water, and dried to a constant weight at 105°C. The percentage of keratin-biodegradation was determined by calculating the differences between the residual dry weights of a treated sample and its corresponding control (keratin without bacterial inoculation). The release of sulfhydryl groups in the culture medium was estimated at 412 nm using DTNB according to the method of Ellman [28]. Sulphur containing amino acids (cysteine, cystine and methionine) and other free amino acids were detected by HPLC using an analytic Eurospher 100-5 C18 Column (300 mm×4.6 mm) (Knauer, Berlin, Germany) equilibrated at a flow rate of 0.5 ml.min^−1^, with a mobile phase consisting of 50% (v/v) acetonitrile in sodium acetate 50 mM (pH 5.1). The separation was monitored with a variable wavelength U.V. detector operated at 254 nm.

### 2.12. Hide-Dehairing Function of KERUS

A piece of skin with hair (about 6 cm×6 cm) from rabbit, sheep, goat and bovine, and a white chicken feather were placed into 20 ml and 10 ml of buffer B containing a purified KERUS having approximately 2,000 U.ml^−1^ of keratinase, respectively. After 10 h of incubation at 37°C, the skin was taken out and the hair was gently hand-pulled to test whether it had parted from the skin. To the authors’ knowledge, no quantitative method is currently available for determining the dehairing effect, which was, consequently, defined as “no”, “yes” or “easily”. Dehairing efficiency was assessed according to the depilated area of the skin at the end of the process, and the quality of the dehaired skin was estimated according to naked eye and microscopic examinations made after 10 h of treatment. The dehaired skin with high quality showed clean hair pore, clear grain structure, and no collagen damage.

The handling of the skin from rabbit, goat, bovine and sheep as well as feathers animals were carried out in strict accordance with the recommendations in the Guide for the Care and Use of Laboratory Animals issued by the University of Sfax, Tunisia. The protocol was approved by the Tunisian Committee on the Ethics of Animal Experiments.

### 2.13. Molecular Cloning and Expression of the *kerus* Gene

The plasmid DNA preparation, digestion with restriction endonucleases, and fragment separation by agarose gel electrophoresis were performed using general molecular biology techniques as described by Sambrook et al. [21]. Two oligonucleotides were synthesized, based on the high degree of sequence homology published for the keratinase *kerUS* gene of *Bacillus* strains, and used for the isolation and determination of the *kerUS* encoding gene sequence. The complete *kerUS* gene and its flanking regions were amplified using the forward primer F-US (5′-TTAAACTGAAAATACAGAATAATC-3′) and the reverse primer R-US (5′-CCGCTGCCTTTTTCATTTTTTCG-3′) to generate an approximately 1.3 kb PCR fragment using genomic DNA from *Br. brevis* strain US575 as a template and DNA polymerase from *Pyrococcus furiosus* (Biotools, Madrid, Spain).

DNA amplification was carried out using the Gene Amp® PCR System 2700 (Applied Biosystems, Foster City, CA, USA). The amplification reaction mixtures (50 µl) contained 20 pg of each primer, 200 ng of DNA template, amplification buffer, and 2 U of DNA polymerase. The cycling parameters used were 94°C for 5 min, followed by 35 cycles of 94°C for 30 s denaturation, 54°C for 60 s primer annealing, and 72°C for 120 s extension. The PCR products were then purified using an agarose gel extraction kit (Jena Bioscience, GmbH, Germany). The purified 1.3 kb PCR fragment was cloned in pCR-Blunt cloning vector into *E. coli* BL21 [F^–^ *ompT gal dcm lon hsdS_B_(r_B_* ^−^ *m_B_* ^−^ *) λ(DE3 [lacI lacUV5-T7 gene 1 ind1 sam7 nin5*] (Invitrogen, Carlsbad, CA, USA) host strain. Recombinant clones of *E. coli* were grown in LB broth media with the addition of ampicillin (100 µg.ml^−1^), IPTG (160 µg.ml^−1^), and X-gal (360 µg.ml^−1^). A clone was noted to harbor a plasmid called pUS2 and was, therefore, retained for further study. The pUS2 plasmid was digested with *Eco*RI restriction enzyme and used for expression studies. The resulting DNA fragment, which was noted to harbour the *kerUS* encoding gene, was sub-cloned in the pTrc99A vector under the control of the inducible P*tac* promoter that was previously digested with the *Eco*RI restriction enzyme leading to the pUS3 plasmid.

### 2.14. Recombinant Enzyme Localization and Purification

After reaching an optical density of 0.6 at 600 nm, the production of target protein from BL21/pUS3 was induced by the addition of IPTG (4 mM). The keratinase crude extracts were prepared from the extracellular fraction as described in a previous work by Jaouadi et al. [13]. The recombinant enzyme (rKERUS) was purified using the same procedures applied for the purification of the native enzyme.

### 2.15. DNA Sequence Determination and Amino Acid Sequence Alignment

The nucleotide sequences of the cloned 16S rRNA and *kerUS* genes were determined on both strands using BigDye Terminator Cycle Sequencing Ready Reaction kits and the automated DNA sequencer ABI PRISM® 3100-Avant Genetic Analyser (Applied Biosystems, Foster City, CA, USA). Cycle sequencing involved successive rounds of denaturation, annealing and extension in a thermocycler to create a linear amplification of extension products. Dye terminator labeling involved the tagging of each dideoxy terminator with a different fluorescent dye. The RapidSeq36_POP6 run module was used, and the samples were analyzed using the ABI sequencing analysis software v. 3.7 NT. All sequencing data were assembled using the STADEN (version 4.5; http://www.mrclmb.cam.ac.uk/pubseq) and DNASTAR (DNASTAR Inc., Madison, WI, US) software packages.

Multiple nucleotide sequence alignment was performed using the BioEdit version 7.0.2 software program. The amino acid sequence homology was analyzed using the BLASTP program available at the NCBI server to search for similar sequences in the databases. Alignment of protein sequences were carried out using CLUSTALW program at the European Bioinformatics Institute server (http://www.ebi.ac.uk/clustalw).

### 2.16. Statistical Analysis

All data were analyzed using Microsoft Excel. Values are expressed as means ± standard deviation of results from three independent experiments. Data were considered as statistically significant for *P* values of 0.05 or less.

### 2.17. Nucleotide Sequence Accession Number

The data reported in this work for the nucleotide sequences of the 16S rRNA and *kerUS* genes have been submitted to the DDBJ/EMBL/GenBank databases under accession nos. **KC152965** and **KC152966**, respectively.

## Results and Discussion

### 3.1. Screening of Keratinase-Producing Strains

Seventy five bacterial strains that were newly isolated from contaminated soil samples from the SO. SA. CUIR leather tannery of M’Saken (Sousse, Tunisia) were identified as keratinase producers based on their clear zone formation patterns on keratin-containing media at pH 7.4. The ratio of the clear zone diameter and that of the colony served as an index for the selection of strains with high keratinase production ability. Six isolates exhibited a ratio that was higher than 3, with the highest ratio being 5. Strain US575 exhibited the highest extracellular keratinase activity (about 7,500 U.ml^−1^) after 28 h of incubation in an optimized medium and was, therefore, retained for all subsequent studies.

### 3.2. Identification and Molecular Phylogeny of the Microorganism

The identification of the newly isolated bacterium (US575) was based on both catabolic and molecular methods. According to the methods described in the Bergey’s Manual of Systematic Bacteriology, morphological, biochemical, and physiological characteristics, showed that the US575 isolate appeared in a bacillus form and was an aerobic, endospore-forming, Gram-positive, catalase-positive, oxydase-positive, motile and aerobic rod-shaped bacterium. The carbohydrate profile of the isolate was also investigated using API 50 CH strips. The results indicated that while the microorganism could utilize d-glucose, d-fructose, maltose, glycerol, d-mannitol, and d-ribose, it could not utilize d-mannose, d-tagatose, l-arabinose, *myo*-inositol, raffinose, erythritol, and adonitol. All the data obtained with regard to the physiological and biochemical properties of the isolate, therefore, strongly confirmed that strain US575 belonged to the *Brevibacillus* genus.

**Figure 1:**
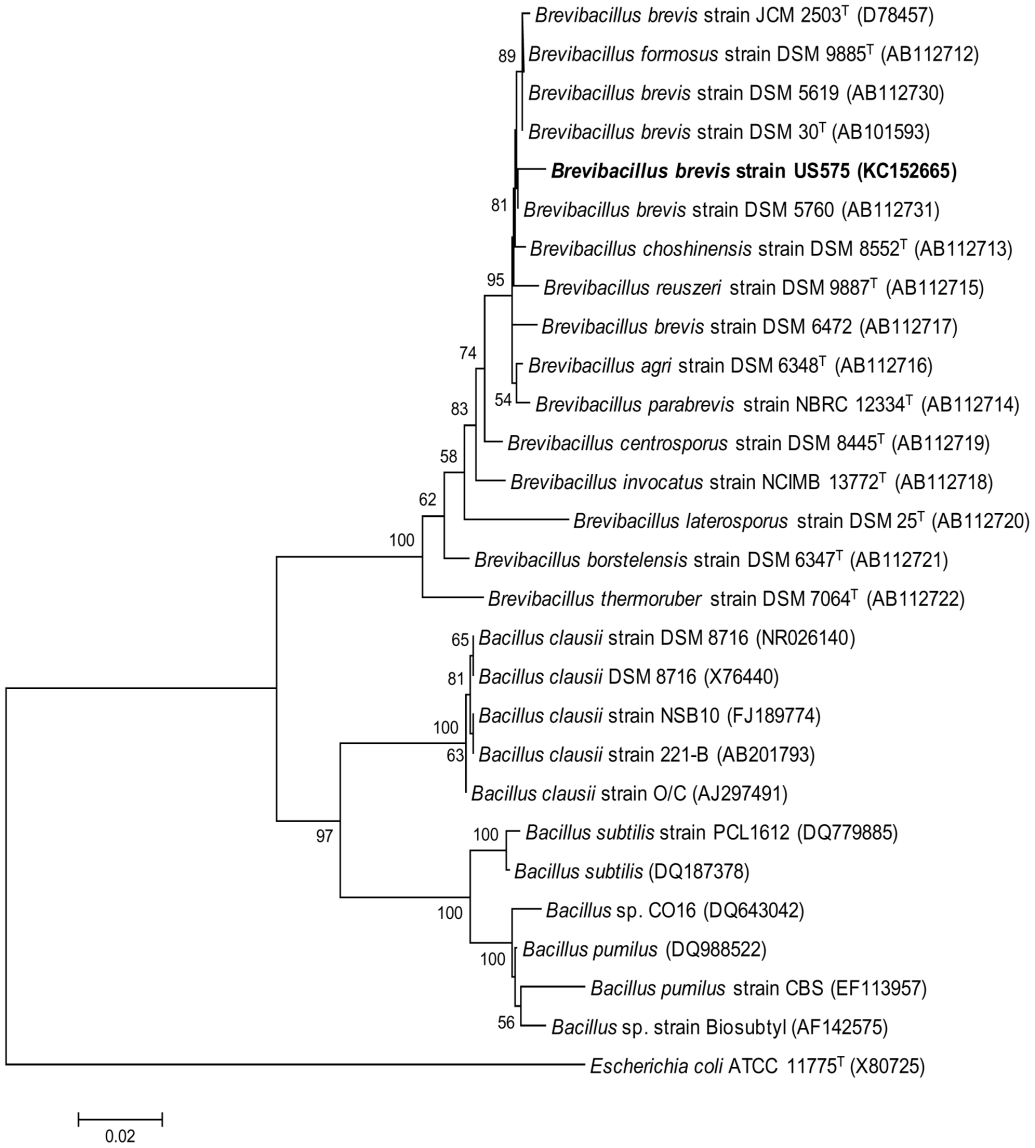
Phylogenetic tree based on 16S rRNA gene sequences within the radiation of the genus *Bacillus*. The sequence of *E. coli* ATCC 11775^T^ (X80725) was chosen arbitrarily as an outgroup. Bar, 0.02 nt substitutions per base. Numbers at nodes (>50%) indicate support for the internal branches within the tree obtained by bootstrap analysis (percentages of 100 bootstraps). NCBI accession numbers are presented in parentheses.

In order to establish further support for the identification of the US575 isolate, a 1506 bp fragment of the 16S rRNA gene was amplified from the genomic DNA of the isolate, cloned in the pGEM-T Easy vector, and sequenced on both strands. The 16S rRNA gene sequence obtained was subjected to GenBank BLAST search analyses, which yielded a strong homology with those of several cultivated strains of *Bacillus*, reaching a maximum of 99% sequence identity. The nearest *Bacillus* strains identified by the BLAST analysis were the *Br. brevis* strain DSM 30^T^ (accession no. AB101593), *Br. brevis* strain DSM 5760 (accession no. AB112731), and *Br. brevis* strain DSM 5619 (accession no. AB112730). Those sequences were imported into the MEGA software package version 4.1 and aligned. Phylogenetic trees were then constructed (Fig. 1), and the findings further confirmed that the US575 strain (accession no. **KC152965**) was closely related to those of the *Brevibacillus brevis* strains. In brief, all the results obtained strongly suggested that this isolate should be assigned as *Brevibacillus brevis* strain US575.

### 3.3. Purification Procedure of KERUS

**Figure 2:**
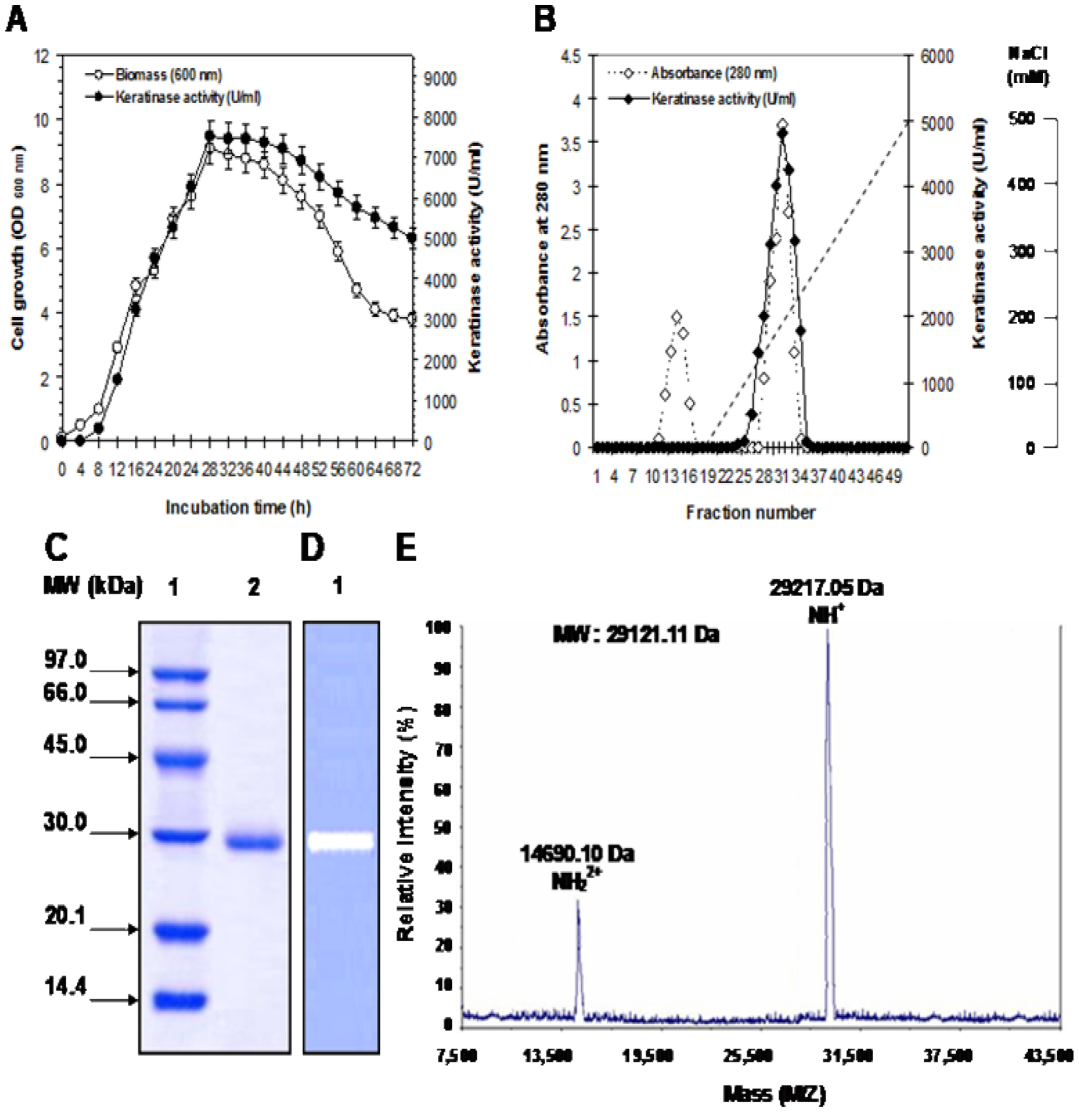
Kinetic production and purification of KERUS. (A) Time course of *Br. brevis* strain US575 cell growth (•) and KERUS production (○). Cell growth was monitored by measuring the OD at 600 nm. (B) Chromatography of the keratinase from *Br. brevis* US575 on Mono Q Sepharose. The column (2.6 cm×20 cm) was equilibrated with buffer C. Adsorbed material was eluted with a linear NaCl gradient (0 mM to 500 mM in buffer C) at a flow rate of 40 ml.h^−1^, and assayed for protein content at 280 nm (◊) and keratinase activity (♦) as described in Section 2. (B) SDS-PAGE of the purified keratinase. Lane 1, protein markers. Lane 2, purified KERUS (30 µg) obtained after Q Sepharose cation-exchange chromatography (fractions 26–34), (D) Zymogram activity staining of the purified keratinase, and (E) MALDI-TOF spectrum of 10 pmol purified KERUS from *Br. brevis* US575. The mass spectrum shows a series of multiply protonated molecular ions. The molecular mass of the enzyme was found to be 29121.11 Da.

The supernatant was obtained by the centrifugation of a 28-h culture of the *Br. brevis* strain US575 (Fig. 2A) using broth (500 ml) as a crude enzyme solution. The enzymatic preparation was precipitated between 50% and 70% ammonium sulphate saturation. The precipitate formed was collected by centrifugation, dissolved in a minimum amount of buffer B, and then dialyzed overnight against repeated changes of the same buffer. Fractions corresponding to keratinase activity were pooled, and then loaded on an HPLC Bio-Sil SEC 125-5 column equilibrated with buffer B. Purification to homogeneity was achieved using Mono Q Sepharose cation-exchange chromatography. Bound proteins were eluted with a linear gradient of NaCl from 0 mM to 500 mM in buffer C at a rate of 40 ml.h^−1^. The protein elution profile obtained at the final purification step indicated that the keratinase was eluted at 140–240 mM NaCl (Fig. 2B). The results of the purification procedure are summarized in Table 1.

**Table 1:** Flow sheet for KERUS purification.

Purification step	Total activity (units)a,b×10^3^	Total protein (mg)a,c	Specific activity(U.mg^−1^ of protein)^a^	Activity recovery rate (%)	Purification factor (fold)
Crude extract	3750±35	6045±75	620±102	100	1
(NH_4_)_2_SO_4_Fractionation (50%–70%)	3225±21	631±30	5110±350	80	8.24
HPLC (Bio-Sil SEC 125-5)	1967±18	172±6	11436±517	61	18.44
Mono Q Sepharose	511±11	27±2	21086±760	26	34.01

a The experiments were conducted three times and ± standard errors are reported.

b One keratin unit is defined as an increase of 0.1 absorbance at 440 nm per minute, using keratin azure as a substrate under the experimental conditions used.

c Amounts of protein were estimated by the method of Bradford [21].

Enzyme purity was estimated to be about 34.01-fold greater than that of the crude extract. Under optimum assay conditions, the purified enzyme had a specific activity of 21,086 U.mg^−1^, with a yield of about 26%. This preparation was a homogeneous enzyme with high purity for it exhibited a unique symmetrical elution peak with a retention time of 18 min, corresponding to a protein of nearly 29 kDa on HPLC gel filtration chromatography (data not shown). SDS-PAGE analysis showed that the pooled fractions displayed one band corresponding to an apparent molecular mass of about 29 kDa (Fig. 2C). Zymogram activity staining revealed one zone of keratinolytic activity for the purified sample co-migrating with proteins of an estimated molecular mass of 29 kDa (Fig. 2D). Results from MALDI-TOF/MS analysis confirmed that the purified SAPDZ had an exact molecular mass of 29121.11 Da (Fig. 2E). Taken together, these observations strongly suggested that KERUS was a monomeric protein comparable to those previously reported for other keratinases from *Bacillus* strains [12], [13], [29], [30], [31], [32].

### 3.4. N-terminal Amino Acid Sequence Determination

The sequence determined for the first 27 N-terminal amino acid of KERUS from the *Br. brevis* strain US575, AQTVPYGIPQIKEPAVHAQGYKGANVK, showed uniformity, thus indicating that it was isolated in a pure form. This sequence was submitted to comparisons with existing protein sequences in the GenBank non-redundant protein database and the Swiss-Prot database (http://www.expasy.ch/sprot/), using the BLASTP and tBlastn search programs. The sequence showed homology with those found for other *Bacillus* keratinases, reaching 100% identity with the keratinase from *B. pumilus* A1 (AQTVPYGIPQI, protein_id = ACM47735.1), and 95% identity with the keratinolytic protease from *B. pumilus* CBS (AQTVPYGIPQIKAPAVHAQGY, protein_id = CAO03040.1). It also showed 92% with subtilisin Carlsberg (AQTVPYGIPLIKADK, protein_id = ZP_03052800.1). The N-terminal amino acid of KERUS differed from those of the three keratinases by only one amino acid; the Gln13 residue in KERUS was an Ala13 in the other enzymes.

### 3.5. Effects of Inhibitors and Metal Ions on KERUS Stability

Proteases can be classified based on their sensitivity to various inhibitors [6]. Accordingly, further assays were performed to evaluate the effects that natural and synthetic inhibitors, as well as chelating agents and group-specific reagents, might have on protease activity determined as the molar ratio of inhibitor/enzyme = 100. The findings indicated that enzyme activity was strongly inhibited by PMSF and DFP, which are well-known inhibitors of serine proteases. This suggested that a serine was involved in the catalytic activity. Other inhibitors, such TPCK and TLCK (chymotrypsin alkylating agents), benzamidine and aprotinin (trypsin competitive reagents), and SBTI (soybean trypsin inhibitor), did not display any inhibitory effects. This inhibition profile further confirmed that the extracellular KERUS enzyme belongs to the serine proteases family. Moreover, the thiol reagent (2-ME, ld-DTT, DTNB, NEM, Iodoacetamide, Leupeptin) and acid reagent (Pepstatin A) had almost no effect on enzyme activity. The keratinase was, however, noted to retain 88 and 90% of its activity in the presence of 10 mM EDTA and 2 mM EGTA as metalloprotease inhibitors, respectively, which suggested that no metal cofactors were required for its function. In fact, serine-proteases are known to contain two calcium binding sites and the removal of Ca^2+^ from the strong binding site is associated with a significant decrease in thermal stability. The role of Ca^2+^ is probably related to the stabilization of the activated form of the KERUS and the preservation of its structure against autolysis.

Several metal ions were also assayed for their effects on KERUS activity (Table 2). The activity of the enzyme was essentially unaffected by monovalent cations (Li^+^, Na^+^, and K^+^). Its activity was enhanced by 161%, 190%, and 325%, following the addition of MnCl_2_, MgCl_2_, and CaCl_2_ at 5 mM, as compared to the control, respectively. This result indicated that the enzyme required Ca^2+^, Mg^2+^, and Mn^2+^ for optimal activity. Furthermore, the enzyme was slightly activated by Co^2+^ and Cu^2+^ and underwent no significant inhibition in the presence of Ba^2+^ and Fe^2+^. Keratinase activity was, however, completely inhibited by Hg^2+^, Ni^2+^, and Cd^2+^ and moderately inhibited by Zu^2+^. Likewise, the purified SAPB keratinolytic protease from *B. pumilus* CBS was previously reported to be totally activated by Ca^2+^, Mg^2+^, and Mn^2+^ but strongly inhibited by Cd^2+^, Ni^2+^, and Hg^2+^ (5 mM) [12], [13]. The increase in protease activity with Ca^2+^, Mg^2+^, and Mn^2+^ indicated that the metal ions exerted a protective effect for the enzyme against thermal denaturation, thereby playing a vital role in maintaining its active confirmation at higher temperature [33].

**Table 2:** Effects of various inhibitors, reducing agents, and metal ions on KERUS stability.

Inhibitor/reducing agent/metal ions	Concentration	Residual activity (%)^a^
None	–	100±2.5
PMSF	5 mM	0±0.0
DFP	2 mM	0±0.0
SBTI	3 mg.ml^−1^	101±2.5
TLCK	1 mM	102±2.5
TPCK	1 mM	99±2.5
Benzamidine	5 mM	104±2.5
MIA	50 µM	98±2.5
ld-DTT	10 mM	96±2.5
2-ME	5 mM	99±2.5
DTNB	10 mM	98±2.5
EPNP	5 mM	103±2.5
NEM	2 mM	95±2.4
Iodoacetamide	5 mM	98±2.5
Leupeptin	50 µg.ml^−1^	96±2.4
Pepstatin A	5 µg.ml^−1^	102±2.5
EDTA	10 mM	89±2.1
EGTA	2 mM	91±2.2
Ca^2+^ (CaCl_2_)	5 mM	325±7.1
Mg^2+^ (MgCl_2_)	5 mM	190±4.3
Mn^2+^ (MnCl_2_)	5 mM	161±3.1
Cu^2+^ (CuCl_2_)	5 mM	130±2.8
Zn^2+^ (ZnCl_2_)	5 mM	119±4.2
Co^2+^ (CoCl_2_)	5 mM	113±2.7
Ba^2+^ (BaCl_2_)	5 mM	97±2.5
Fe^2+^ (FeCl_2_)	5 mM	94±2.3
Ni^2+^ (NiCl_2_), Cd^2+^ (CdCl_2_), Hg^2+^ (HgCl_2_)	5 mM	0±0.0
Li^+^ (LiSO_4_), K^+^ (KCl), Na^+^ (NaCl)	5 mM	100±2.5

a Values represent the means of three replicates, and ± standard errors are reported.

### 3.6. Effects of pH and Temperature on Keratinase Activity and Stability

**Figure 3:**
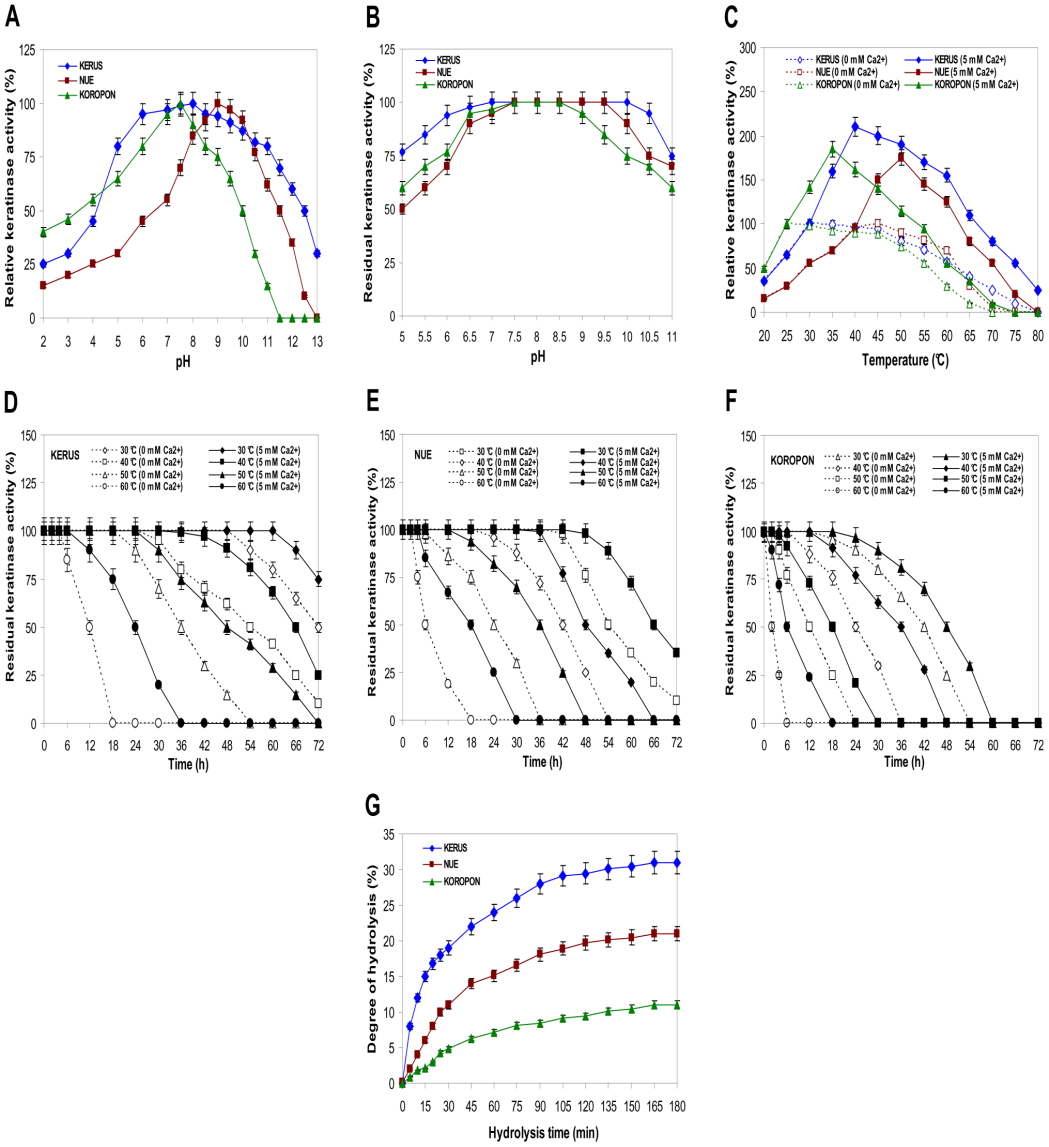
Effects of pH and temperature on the activity and stability of KERUS, NUE, and KOROPON. Effects of pH on the activity (A) and stability (B) of KERUS, NUE, and KOROPON. The activity of the enzyme at optimum pH was taken as 100%. Buffer solutions used for pH activity and stability are presented in Section 2. Effects on the thermoactivity (C) and the thermostability of KERUS (D), NUE (E), and KOROPON (F). The enzyme was pre-incubated in the absence or presence of CaCl_2_ at various temperatures ranging from 30°C to 60°C. Residual protease activity was determined from 0 h to 72 h at 6 h intervals. The activity of the non-heated enzyme was taken as 100%. (G) Hydrolysis curves of chicken feather-meal proteins treated with various purified enzymes. The purified proteases used were: KERUS (♦), NUE (▪), and KOROPON (Δ). Each point represents the mean (n = 3) ± standard deviation.

Fig. 3A shows that KERUS, NUE, and KOROPON displayed activities over a broad range of pH (3–11), with an optimum at pH 8, 9, and 7.5, respectively. The relative activities at pH 10 were 50%, 80%, and 85% for NUE, KERUS, and KOROPON, respectively. The pH stability profile indicated that the purified enzymes were highly stable in the pH range between 5 and 10 (Fig. 3B). KOROPON, KERUS, and NUE retained 61%, 75%, and 82% of their activity at pH 5 after 72 h incubation at 40°C, respectively.

At pH 8, and using keratin azure as substrate, KERUS, NUE, and KOROPON were optimally active at 30°C, 45°C and 25°C in the absence of CaCl_2_ and at 40, 50 and 35°C in the presence of 5 mM Ca^2+^, respectively (Fig. 3C). The half-life times of KERUS in the absence of additives were 72 h, 54 h, 36 h, and 12 h at 30°C, 40°C, 50°C, and 60°C, respectively (Fig. 3D). As shown in Fig. 3D, the half-life times of KERUS at 30, 40 50, and 60°C increased to 80 h, 66 h, 48 h, and 24 h in the presence of 5 mM CaCl_2_, respectively. In fact, Ca^2+^ was previously reported to improve the activity and stability of the *B. pumilus* CBS protease [13]. The findings indicate, however, that the thermoactivity and thermostability of KERUS were higher than those recorded for NUE and KOROPON as well as those previously reported for several other proteases from *Bacillus* [13], [14], [34], [35], [36].

### 3.7. Effect of Substrate Specificity and Determination of Kinetic Parameters

The substrate specificity of proteases is often attributed to the amino acid residues preceding the peptide bond they hydrolyze. The relative hydrolysis rates of various substrates were investigated to elucidate the amino acid preference/substrate specificity of KERUS (Table 3). The highest activity was observed with keratin, keratin azure, gelatin, elastin, and casein. A relatively high activity against myoglobin and albumin was also observed. No collagenase activity was detected on collagen types I and II, which provides further support for the utility of KERUS for hair removal in the leather industry. The lack of collagenase activity is an advantage in the leather industry because collagen, the major leather-forming protein, would not be significantly degraded. This criterion was satisfied by KERUS, which makes it suitable for the dehairing of animal hides.

**Table 3:** Effect of substrate specificity on KERUS activity.

Substrate	Concentration	Monitoring wavelength (nm)^a^	Relative activity (%)^b^
*Natural protein*			
Keratin	30 g.l^−1^	660	100±2.5
Gelatin	30 g.l^−1^	660	96±2.4
Elastin-orcein	30 g.l^−1^	660	85±2.1
Casein	30 g.l^−1^	660	80±2.1
Hemoglobin	30 g.l^−1^	660	60±1.6
Myoglobin	30 g.l^−1^	660	51±1.4
Albumin	30 g.l^−1^	660	25±1.1
*Modified protein*			
Keratin azure	30 g.l^−1^	440	100±2.5
Azo-casein	30 g.l^−1^	440	92±2.3
Collagen type I	10 mg.ml^−1^	490	0±0.0
Collagen type II	10 mg.ml^−1^	490	0±0.0
*Ester*			
BAEE	15 mM	253	100±2.5
BCEE	15 mM	253	91±2.4
BTEE	15 mM	253	0±0.0
ATEE	15 mM	253	0±0.0
*Synthetic peptide*			
Suc-Tyr-Leu-Val-*p*NA	5 mM	410	100±2.5
Suc-(Ala)_2_-Phe-*p*NA	5 mM	410	94±2.4
Suc-(Ala)_3_-*p*NA	5 mM	410	80±2.1
BAPNA	5 mM	410	75±2.0
Suc-(Ala)_2_-Val-*p*NA	5 mM	410	70±1.9
Suc-(Ala)_2_-Val-Ala-*p*NA	5 mM	410	61±1.6
Met Suc-(Ala)_2_-Pro-Val-*p*NA	5 mM	410	25±1.1
Suc-(Ala)_2_-Pro-Met-*p*NA	5 mM	410	20±1.0
Suc-(Ala)_2_-Pro-Phe-*p*NA	5 mM	410	15±0.9
Suc-(Ala)_2_-Pro-Leu-*p*NA	5 mM	410	12±0.8
Glu-(Ala)_2_-Pro-Leu-*p*NA	5 mM	410	11±0.7
Suc-Ala-Pro-Ala-*p*NA	5 mM	410	10±0.7

a Values represent means of three replicates, and ± standard errors are reported.

b The activity of each substrate was determined by measuring absorbance at specified wavelengths according to the relative method reported [10].

The purified KERUS was noted to exhibit esterase and amidase activities on BAEE, BCEE, and BAPNA, but not on BTEE and ATEE. It also displayed a preference for aromatic and hydrophobic amino acid residues, such as Phe, Leu, Ala, and Val, and the carboxyl side of the splitting point in the P1 position. KERUS was, therefore, active against leucine peptide bonds, a quality that was previously demonstrated for KERAB [10] and SAPDZ [15]. When Suc-(Ala)_n_-*p*NA was used as the synthetic oligopeptide substrate, a minimum length of two residues was required for hydrolysis. Enzymatic activity was noted to largely depend on secondary enzyme substrate contacts with amino acid residues (P2, P3, etc.) more distant from the scissile bond, as illustrated by the differences observed between the kinetic parameters of Suc-Tyr-Leu-Val-*p*NA and Suc-(Ala)_2_-Val-Ala-*p*NA. The highest hydrolysis rates recorded for KERUS were 100% and 94%, which were attained with Suc-Tyr-Leu-Val-*p*NA and Suc-(Ala)_2_-Phe-*p*NA, respectively. Its preference for lager hydrophobic amino acids could presumably be due to the active site cleft or the crevice lined with hydrophobic amino acids residues.

KERUS, NUE, and KOROPON exhibited the classical kinetics of Michaelis-Menten for the four substrates used. The order of the catalytic efficiency (*k*_cat_/*K*_m_) values of each enzyme was almost the same, *i.e.*, Suc-Tyr-Leu-Val-*p*NA>BAEE>keratin azure>keratin (Table 4). When keratin was used as a protein substrate, the *k*_cat_/*K*_m_ exhibited by KERUS were 1.85 times and 2.78 times higher than those of NUE and KOROPON, respectively. When Suc-Tyr-Leu-Val-*p*NA was used as a synthetic substrate, KERUS was also noted to exhibit *k*_cat_/*K*_m_ values that were 2.81 times and 3.95 times higher than those of NUE and KOROPON, respectively (Table 4).

**Table 4:** Kinetic parameters of purified keratinolytic proteases: KERUS, NUE, and KOROPON for the hydrolysis of protein, ester, and synthetic peptide substrates.

Substrate	Enzyme	*K*_m_ (mM)	*V_max_* (×10^3^ U.mg^−1^)	*k*_cat_ (×10^3^ min^−1^)	*k*_cat_/*K*_m_(×10^3^ min^−1 ^mM^−1^)	Catalytic efficiency relative to KERUS
Keratin	KERUS	0.513±0.012	21.150±470	14.100	27.485	1.00
	NUE	0.698±0.036	15.580±315	10.386	14.879	0.54
	KOROPON	0.745±0.042	11.050±168	7.366	9.887	0.35
Keratin azure	KERUS	0.480±0.009	13.431±125	8.954	18.654	1.00
	NUE	0.736±0.040	8.944±114	5.962	8.100	0.43
	KOROPON	0.849±0.054	5.210±095	3.473	4.090	0.21
BAEE	KERUS	0.655±0.033	33.542±610	22.361	34.138	1.00
	NUE	0.917±0.058	22.106±505	14.737	16.070	0.47
	KOROPON	1.010±0.062	18.685±432	12.456	12.332	0.36
Suc-Tyr-Leu-Val-*p*NA	KERUS	0.805±0.048	52.250±915	34.833	43.270	1.00
	NUE	1.303±0.067	30.009±604	20.006	15.353	0.35
	KOROPON	1.425±0.071	23.411±516	15.607	10.952	0.25

Values represent the means of three replicates, and ± standard errors are reported.

### 3.8. Determination of Hydrolysis Degree

The hydrolysis curves of chicken feather-meal protein after 4 h of incubation are shown in Fig. 3G. The purified enzymes were used at the same levels of activity (2,000 U.ml^−1^) for the production of protein hydrolysates from chicken feather-meal and for the subsequent comparisons of hydrolytic efficiencies. The chicken feather-meal was noted to attain high hydrolysis rates during the first 1 h. The enzymatic reaction was then noted to decrease and to reach a subsequent steady-state phase where no apparent hydrolysis took place. As shown in Fig. 3G, the purified KERUS was the most efficient (31%) keratinase used during hydrolysis, flowed by NUE (21%), with KOROPON being the least efficient (11%) one. These findings indicate that KERUS can be useful for upgrading the nutritional value of feather-meals.

### 3.9. The Keratin-Biodegradation Profile of *Br. Brevis* Strain US575

**Figure 4:**
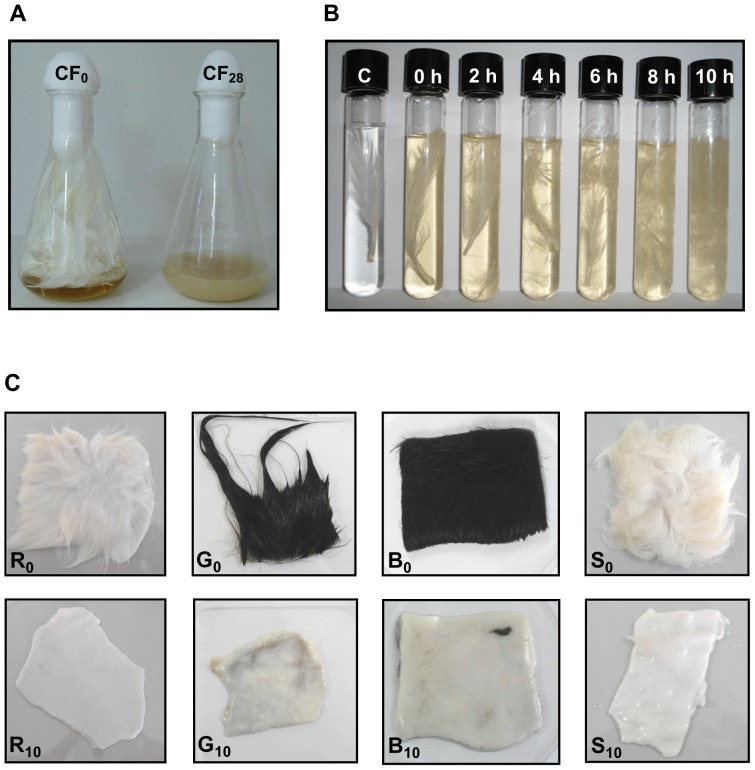
Keratin(feather)-biodegradation by *Br. brevis* US575 and hide-dehairing function of KERUS. (A) Feathers were incubated for 28 h at 37°C under shake culture condition with 3.5×10^7^ cells ml^−1^ as an initial inoculum density for the US575 strain (right flask, t = 28 h) and with autoclaved inoculum as control (left flask, t = 0 h). (B) KERUS was incubated for 10 h at 37°C with feather, C = control. (C) KERUS was incubated for 10 h at 37°C with rabbit hair (R_0_: rabbit hair as control, t = 0 h; R_10_: rabbit hair treated, t = 10 h), (G_0_: goat hair as control, t = 0 h; G_10_: goat hair treated, t = 10 h), (B_0_: bovine hair as control, t = 0 h; B_10_: bovine hair treated, t = 10 h), (S_0_: sheep wool as control, t = 0 h; S_10_: sheep wool treated, t = 10 h).

The *Br. brevis* strain US575 was able to grow in the initial medium containing 10 g.l^−1^ of chicken feather-meal, chicken feather (Fig. 4A), rabbit hair, goat hair, bovine hair, and sheep wool (as sole carbon, nitrogen, and energy sources), reaching absorbance values ranging between 8 and 11.5 at 600 nm after 28 h of culture. Of the 6 keratin substrates tested, feather-meal was the most strongly degraded (100%), followed by chicken feather (93%), rabbit hair (86%), goat hair (77%), bovine hair (66%), and sheep wool (12%). The feather-meal degradation rate displayed by *Br. brevis* strain US575 was higher than those previously reported for *B. pumilus* CBS [20] and *B. pumilis* F3–4 (97%) [37].

Furthermore, the maximum protein release rates obtained for the *Br. brevis* strain US575 were in the feather-meal medium, followed by chicken feather. Feather-meal and chicken feather also gave the best KERUS production yields of 7,500 U.ml^−1^ and 6,300 U ml^−1^ respectively, whereas sheep wool supported very low keratinolytic activity (1,875 U ml^−1^) (Table 5). Full-grown and intense feather-biodegradation activity could, therefore, be achieved in 28 h of incubation at 40°C and pH 8 (Fig. 4A). This profile contrasts with the ones previously reported for *B. pumilus* CBS which solubilized feather in 24 h at pH 8–9 and 30–37°C [24], *B. pumilus* FH9 which solubilized feather in 72 h at pH 9 and 55°C [38], and *B. pumilis* F3–4 which showed intense feather-biodegradation activity in 168 h at pH 7.5 and 30°C [37].

**Table 5:** Effect of keratinacious substrates on the keratinase KERUS of *Br. brevis* strain US575 after 28 h of incubation.

Keratinacious substrates	Soluble protein (mg.ml^−1^)	keratinolytic activity (U.ml^−1^)	SH group ( µM)	Keratin-biodegradation (%)
Feather-meal	6.11	7500	7.20	100
Chicken feather	5.40	6300	6.52	93
Rabbit hair	4.80	5500	5.16	86
Goat hair	4.01	3135	4.01	77
Bovine hair	2.95	2150	3.10	66
Sheep wool	1.54	1875	1.13	12

Values represent the means of three replicates, and ± standard errors are reported.

The levels of proteins and sulfhdryl groups were noted to increase concurrently with the increase of keratin-biodegradation (Table 5). Higher degrees of keratin-biodegradation resulted in higher levels of sulfhydryl group formation. The results, therefore, suggested that *Br. brevis* US575 had a disulfide bond-reducing ability. The data from amino acid analysis following keratin-biodegradation also revealed a marked increase in the release of free amino acids after 10 h of incubation. The profile suggests that phenylalanine, tryptophan, isoleucine, leucine, valine, and alanine were the major amino acids liberated, whereas untreated keratin (control) did not release any free amino acids. The amino acid profile of KERUS matched well with the ones previously reported for the keratinolytic proteases produced by *B. pumilus* CBS [24], *B. circulans* DZ100 [15], and *B. licheniformis* PWD-1 [39].

### 3.10. Hide-Dehairing Ability of KERUS

A partial degradation was observed to occur simultaneously with an increase in protein concentration and sulfhydryl group formation after 10 h of shaking-incubating KERUS enzyme with a white feather; no degradation was, however, noted with the control (Fig. 4B). Barbules and rachises were completely degraded to fine granule forms at the bottom of the test tubes. The incubation of KERUS with skin from rabbit, goat, bovine and sheep for dehairing showed that after 10 h of incubation at 37°C, all skins had their hairs removed very easily as compared to their corresponding controls, with no observable damage on the collagen (Fig. 4C). The dehaired skins were, therefore, noted to display clean hair pores and clear grain structures. These findings provided evidence that KERUS, alone, could accomplish the whole process of dehairing. In leather processing, the dehairing function is generally carried out under a relatively high pH value that ranges between 8 and 10 [40], and KERUS was noted to meet this criterion. Relatively similar results were previously attained by the *A. tamarri* alkaline protease on goat skin at pH 9–11 and 30–37°C [40]. The *Vibrio sp*. Kr2 strain was, however, reported to achieve the same result but at pH 6–8 and 30°C [41]. Alkaline proteases from *B. pumilus* were also reported to have high keratinolytic activity and to accomplish the dehairing process on their own for bovine hair [34], cowhides [42], and goatskins [35]. In this respect, and showing higher feather-biodegradation and dehairing abilities, KERUS may be considered a potential promising candidate for future application in biotechnological bioprocesses involving the dehairing of hides or skins and the conversion of feather-rich wastes into economically useful feather-meal. Accordingly, further studies, some of which are currently underway, are needed to test the hide and skin dehairing potential of KERUS at a semi-industrial scale in the leather processing industry.

### 3.11. Cloning and Sequencing of the *kerus* Gene

Using the keratinase gene sequences of *Bacillus* strains, two primers, called F-US and R-US, were designed and used to amplify a fragment of about 1.3 kb that could contain the *kerUS* gene. This PCR fragment was purified and cloned in a pCR-Blunt cloning vector using an *E. coli* BL21 host strain, thus leading to pUS2.

**Figure 5:**
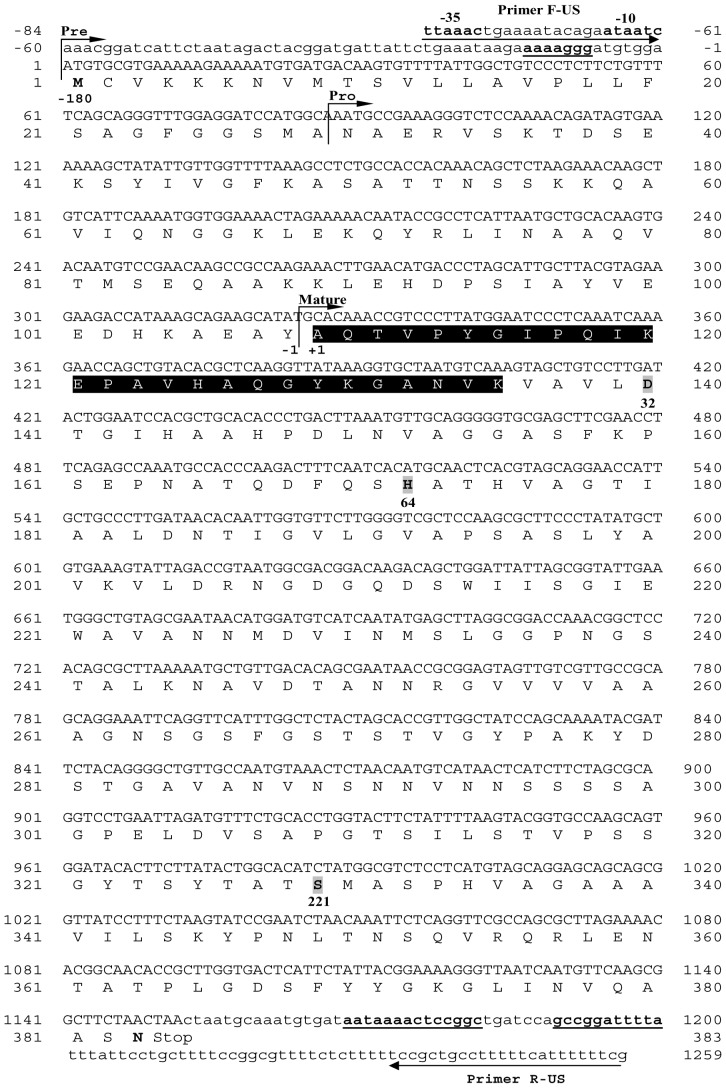
Nucleotide and deduced amino acid sequences of the *kerUS* gene. The *kerUS* consisted of 1149-bp encoding a polypeptide of 383 aa residues. The putative starting residues of the pre-protein (pre), pro-protein (pro), and mature protein (mature) are indicated. The nucleotide and predicted amino acids are numbered on the right and on the left. The inverted arrows indicate the position of the primers F-US and R-US. The catalytic center is indicated in bold and grey. The possible Shine-Dalgarno sequence and the transcriptional terminator sequences are bolded and underlined, and the putative −35 and −10 promoters are shown in bold. The black box indicates the N-terminal amino acid sequence of the purified KERUS.

The complete nucleotide sequence of the *kerUS* gene and the amino acid sequence deduced are shown in Fig. 5. The analysis of the nucleotide sequence of the *kerUS* gene and its flanking DNA regions revealed the presence of an open reading frame (ORF) of 1,149-bp that encoded a pre-pro-enzyme consisting of 383 aa with a predicted molecular weight of 39498.08 Da. This ORF started with an ATG codon at nucleotide position 1 and terminated with a TAA stop codon. A Shine-Dalgarno-like sequence was observed 7 bp to 13 bp upstream from the ATG codon. The presumed putative promoter region, −10 (ATAATC) and −35 (TTAAAC) sequences resembled the consensus sequences determined for the promoter region by the lambda P_R_ RNA polymerase of *E. coli*. This ORF was confirmed as the gene encoding KERUS since, as determined by the Edman degradation method, the deduced amino acid sequence was noted to include the 27 N-terminal amino acid sequence of the purified KERUS. This sequence was identical to those of keratinases from other *Bacillus* strains [13], [14], [35], [36].

### 3.12. Amino Acid Sequence Inspection

**Figure 6:**
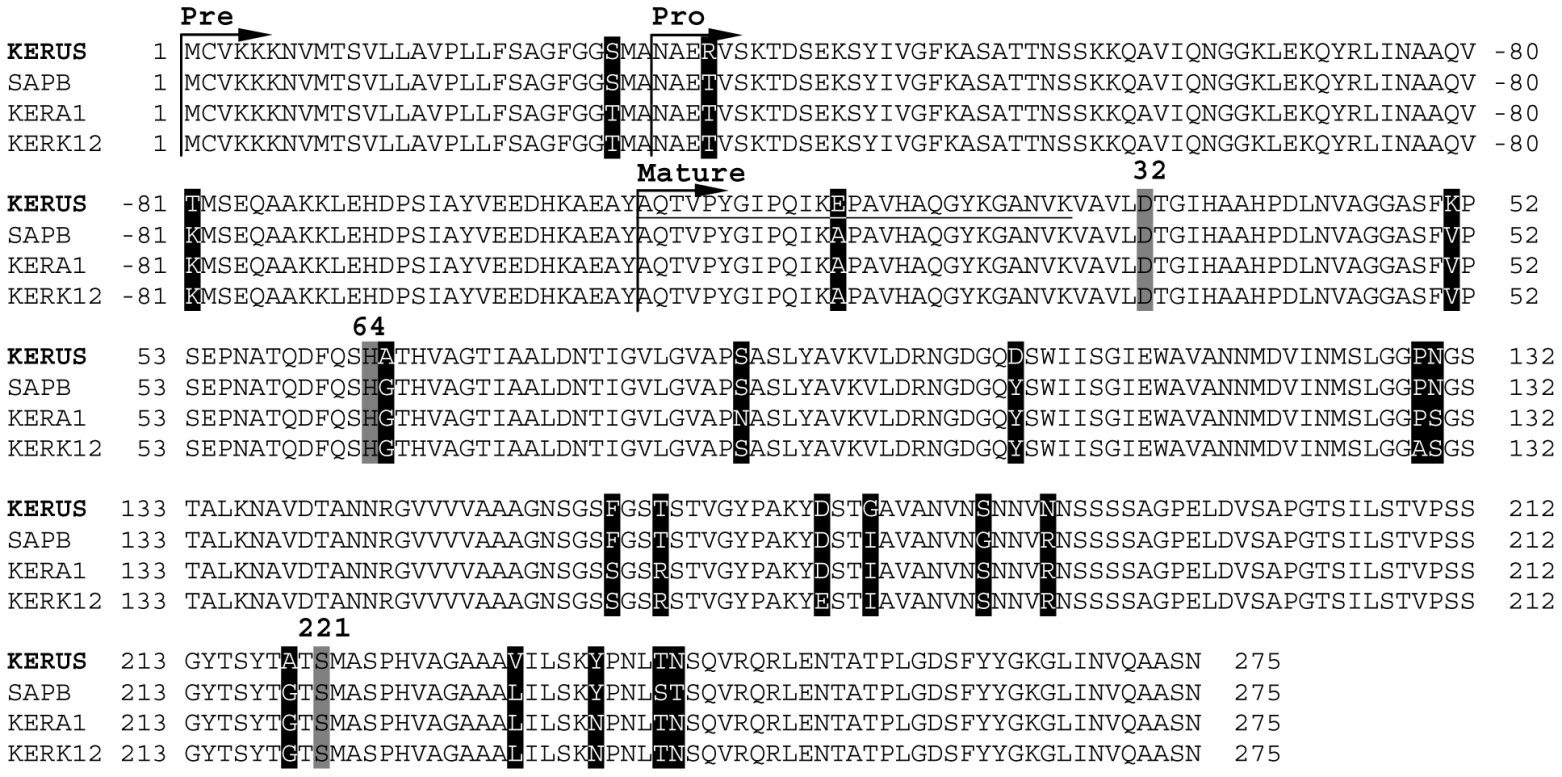
Amino acid sequence alignment of KERUS from *Br. brevis* strain US575 with other *Bacillus* keratinases. The used keratinases are: the SAPB from *B. pumilus* strain CBS, the KERA1 from *B. pumilus* strain A1, and the KERK12 from *B. pumilus* strain KS12. The first amino acid of the mature keratinase, Ala, is counted as +1. The putative starting residues of the pre-peptide (pre), pro-peptide (pro), mature protease, and active site residues D32, H64, and S221 are indicated. X (highlighted character) shows amino acid changes in KERUS with regards to other serine keratinases.

SignalP, version 3.0 (http://www.cbs.dtu.dk/services/SignalP/), predicted a signal peptide (pre-sequence) of 29 aa bordered with the signal peptidase recognition (SPR) site A-N-A, indicating that a group of strongly hydrophobic amino acids was conserved. Belonging to the signal sequence, the pro-sequence consisting of 79 aa had to be cleaved by autoproteolytic processing in the periplasm. The active mature keratinase consisted of 275 aa, with a predicted molecular weight of 27801.67 Da and a predicted isoelectric point of 5.81. The apparent molecular weight of the purified enzyme (29 kDa) determined by SDS-PAGE, MALD-TOF/MS, and HPLC gel filtration chromatography was in good agreement with the predicted value. The typical triad catalytic residues (D32, H64 and S221) in the active site as well as three serine protease signatures (amino acid residues 28–39, 64–75 and 216–226) [43] were also conserved in the *kerUS* gene. The amino acid sequence deduced from the nucleotide sequence of the *kerUS* gene was compared to those of other known keratinases from *Bacillus* strains (Fig. 6).

The classification analysis of the deduced amino acid sequence demonstrated that the mature keratinase was a member of the serine protease family. The alignment of the deduced amino acid sequence of *kerUS* with those of known keratinases revealed high homology with the extracellular serine proteases previously isolated and characterized from *Bacillus* strains. Nevertheless, one amino acid (S/T) in the prepeptide, two amino acids (R/T and T/K) in the propeptide, and 11 aa (E13A, K51V, A65G, D104Y, G175I, S182G, N186R, A219G, V233L, T242S, and N243T), 13 aa (E13A, K51V, A65G, S87N, D104Y, N130S, F159S, T162R, G175I, N186R, A219G, V233L, and Y238N), and 14 aa (E13A, K51V, A65G, D104Y, P129A, N130S, F159S, T162R, D172E, G175I, N186R, A219G, V233L, and Y238N) in the mature KERUS were noted to differ from the SAPB [13], KERA1 [44], and KERK12 [45] residues, respectively (Fig. 6). Although displaying high levels of homology, the latter keratinases exhibited relatively different characteristics. In fact, there were marked differences between their biochemical properties as compared to that of KERUS. The pH and temperature optima shown by SAPB [13], KERA1 [44], and KERK12 [45] were 10.6/65°C, 9/60°C, and 10/60°C, respectively.

### 3.13. Expression of the *kerus* Gene in *E. Coli* and Characterization of the Recombinant Enzyme

To express KERUS, the corresponding gene was cloned downstream of P*T7* or P*tac* promoters in pUS2, and pUS3, respectively, and then introduced to the BL21 strain. The intracellular and periplasmic fractions of all recombinant strains displayed no alkaline protease activity. Relatively high specific activities of 3,250 U mg^−1^ and 20,000 U mg^−1^ were, however, detected in the extracellular fractions of BL21/pUS2, and BL21/pUS3, respectively. Based on this study, KERUS was most efficiently expressed with the P*tac*-*kerUS* construction (pUS3), which was, therefore, retained for the purification of the recombinant keratinase (rKERUS). The extracellular rKERUS was purified using the same strategy employed for the native enzyme from *Br. brevis* strain US575. All the biochemical characteristics identified from rKERUS were almost similar to those of the original enzyme. The large-scale preparation of rKERUS as a biocatalyst for biotechnological application can, therefore, be easily performed and may offer new promising opportunities for the enhancement of several biotechnological bioprocesses, particularly in the leather and poultry processing industries.

## Conclusions

The extracellular keratinase (KERUS) from *Br. brevis* US575 was purified and biochemically characterized. The nucleotide sequence of the *kerUS* gene and its flanking regions were determined and expressed in *E. coli*. The results revealed that KERUS has powerful abilities for the biodegradation of chicken feather-meal and the dehairing of various skins with minimal damage to collagen. The enzyme also showed a number of properties that are highly valued in the poultry and leather processing industries. Overall, the findings indicated that KERUS could be considered a potential promising candidate for application in the dehairing of skins and hides in the leather processing industry as a substitute to the currently employed toxic chemicals. Accordingly, further studies, some of which are currently underway in our laboratories, are needed to explore the structure-function relationships of the enzyme using site-directed mutagenesis and 3-D structure modeling.
